# Verbal working memory performance and proactive interference are largely unaffected by matching font color reinstatement

**DOI:** 10.3389/fpsyg.2025.1688942

**Published:** 2025-11-14

**Authors:** Sara B. Festini

**Affiliations:** Department of Psychology, University of Tampa, Tampa, FL, United States

**Keywords:** encoding specificity, context effects, proactive interference, verbal working memory, reinstatement

## Abstract

The present set of five experiments assessed whether context-dependent memory effects were evident within verbal working memory. Participants completed an item-recognition verbal working memory task at a set size of 4, where test probes were presented in the studied color or in a different color. Experiments 1–3 varied the manner in which color context was implemented. Experiments 4 and 5 additionally induced proactive interference by presenting occasional recent probes that originated from the *prior* trial as opposed to the current trial. Recency-based proactive interference was present but was not influenced by context reinstatement. Indeed, across the 5 experiments and 19 total assessments of context reinstatement, only two statistical tests supported context-dependent working memory facilitation. Instead, the overwhelming majority of evidence indicated that response times, accuracy, and proactive interference were not significantly different when the study/test color contexts matched or mismatched within working memory. Thus, these findings suggest that long-term memory contextual influences are stronger than those operating within working memory for meaningful verbal memoranda.

## 1 Introduction

Have you ever walked into a room with purpose, only to forget your intention? If you walk back to your starting location, you are more likely to remember your goal. Reinstating the study context has often been shown to facilitate memory. The theory of *context-dependent memory*, also referred to as *context reinstatement* or *encoding specificity*, describes the observation that people tend to have better memory when the test context matches the study context (Smith, 2013). One of the most famous examples of encoding specificity documented better memory for words when participants studied and recalled the words both underwater (while scuba diving) or both on land, as opposed to under different study/test contexts (Godden and Baddeley, 1975).

Encoding specificity has been documented with a myriad of different context manipulations, including, for example, physical surroundings (e.g., Bilodeau and Schlosberg, 1952; Smith, 1986; Abernethy, 1940), font type (Arndt, 2010), font or background color (e.g., Sakai et al., 2010; Murnane and Phelps, 1995; Hockley, 2008), mental imagery (Chu et al., 2003), virtual reality (Shin et al., 2021; Essoe et al., 2022), normal versus restricted vision (Dolinsky and Zabrucky, 1983), images/videos on a computer screen (e.g., Hayes et al., 2007; Smith and Manzano, 2010; Isarida et al., 2021), and odors (e.g., Cann and Ross, 1989; Sorokowska et al., 2022). For instance, recognition of studied faces was better when the same odor was present at both the study and test sessions (Cann and Ross, 1989). Word recall was also facilitated when a complex scene video (e.g., of a grocery store) was reinstated at test (Smith and Manzano, 2010). Meta-analyses of context-dependent memory effects find evidence of reliable moderate effect sizes (Smith and Vela, 2001).

Context-dependent memory has most often been studied within long-term memory with long lists and longer delays before memory assessment (e.g., Smith, 1994; Smith, 2013; Smith and Vela, 2001). The present research empirically examines whether encoding specificity also operates strongly within verbal working memory, when a small number of memory representations are currently held in mind and memory is tested after several seconds. Here, working memory is considered to involve the temporary maintenance and use of information held in mind over a short-term delay for current information processing (e.g., Cowan, 2017; i.e., information that one is currently thinking about or “working with” in their mind). Despite slight variations in the conceptual definitions of working memory (see Cowan, 2017), it is generally agreed that working memory is limited in capacity (e.g., Cowan, 2000) and has a heightened state of accessibility for a short duration, often no more than several seconds (e.g., Ricker and Cowan, 2010), unless rehearsal (Baddeley, 1986) or refreshing are implemented (e.g., Camos et al., 2011; Raye et al., 2007). Both time and interference have been proposed to contribute to loss of information from working memory (see Ricker and Cowan, 2010; Oberauer and Lewandowsky, 2008).

Models of working memory account for the processing of both item information and contextual information (such as serial position, pitch, visual color, etc.). For instance, in addition to an attention-controlling central executive, the Baddeley & Hitch Model proposes separate stores for visual and verbal information (i.e., visuospatial sketchpad and phonological loop; Baddeley and Hitch, 1974), as well as an episodic buffer that temporarily binds and stores co-occurring multimodal contextual details into “episodes” within working memory (Baddeley, 2000). Relatedly, the embedded-processes model of working memory (Cowan, 2019; Cowan, 1988; Cowan, 1999) allows for contextually-specific information to be processed in the focus of attention or within the activated long-term memory portion of working memory. Hence, the fact that working memory models include the processing of contextual information suggests that context reinstatement effects are plausible. Nevertheless, such encoding specificity effects have not been thoroughly investigated within verbal working memory tasks.

One prior study implemented a combination of a short-term memory encoding task with long-term memory assessments and an environmental context manipulation. Specifically, Smith (1986) had participants encode words during a short-term memory task (i.e., spoken 5-word sets followed by a 10-s delay before immediate recall) either in an office or cubicle. After a 25-min delay with distraction from two intervening tasks, participants performed long-term memory free recall and forced-choice recognition tests, where the encoding context of an office or cubicle was reinstated or not. Participants exhibited context-dependent memory effects on both delayed tests, although given the lengthy delay with distraction, these assessments are considered long-term memory tests, even though the encoding task was a short-term memory task. Further, importantly, the initial short-term encoding task was performed in the same encoding context for each participant, so context reinstatement was not evaluated for short-term memory performance.

The present research aimed to directly evaluate the effects of context reinstatement within a verbal working memory task. In a series of five experiments, participants studied four words presented simultaneously on a computer screen for several seconds. The words were written in one of four different ink colors (cyan, lime, magenta, or yellow) or displayed upon one of four different colorful backgrounds. After a 3-s delay, a single recognition memory probe was displayed, and participants indicated whether or not the single word was included in the previous memory set as quickly and accurately as possible. Critically, the single probe word was manipulated to appear in a matching color or a mismatching color as during the study episode. In addition to evaluating whether “match” probes were recognized faster or more accurately than “mismatch” probes, the present study also included an assessment of recency-based proactive interference (Experiments 4 and 5) for the memoranda.

Proactive interference (i.e., greater processing difficulty due to familiarity) can be induced by presenting a recognition probe that was included within the prior memory set as opposed to the current memory set (e.g., Atkins et al., 2011; Nee et al., 2007). That is, presenting “recent” (i.e., temporally familiar) probes often induces longer response times and worse accuracy relative to unstudied probe words. An everyday example of proactive interference includes accidentally walking to where you parked your car yesterday instead of where you parked your car today. Here, the color context was manipulated for recent probes to determine if reinstating the color context resulted in heightened proactive interference for Match-Recent as opposed to Mismatch-Recent probes.

Some prior experiments of long-term memory have demonstrated context-dependent interference reduction, such that participants exhibit less memorial interference when studied materials were learned in distinct contexts (Bilodeau and Schlosberg, 1952; Greenspoon and Ranyard, 1957; Strand, 1970). That is, when the contexts were different, there was less interference. However, such context-dependent interference reduction has not always been observed (Nagge, 1936), and these inductions of interference often resulted from a “build up” of overlapping categorical stimulus information (such as presentations of synonyms). Importantly, the present studies did not use overlapping categories, but, instead, manipulated recency-based proactive interference, which has reliably demonstrated lengthened response times for familiar, recent as opposed to novel probes (e.g., Festini, 2020; Festini and Reuter-Lorenz, 2014; Badre and Wagner, 2005; Jonides and Nee, 2006; Nee et al., 2007; Monsell, 1978). Hence, the nature of context reinstatement on verbal working memory recency-based proactive interference induction has yet to be evaluated empirically.

Based on the theories of encoding specificity and context-dependent memory (see Smith, 2013), it is hypothesized that participants will have better memory and faster response times to recognition memory probes that are presented in the same color context during the study and test periods (Match probes) than words that are presented in a different color context (Mismatch probes). Moreover, participants are hypothesized to have greater proactive interference for recent recognition probes presented in the same encoding color context than for recent probes presented in a different encoding color context. Further, given the nature of the rapidly progressing working memory paradigm and lack of provided encoding task (i.e., no instructions to create mental images, sentences, etc.), it is expected that participants are more likely to use more shallow rote rehearsal as opposed to elaborative rehearsal. Shallow processing has been observed to yield stronger context reinstatement effects than elaborative processing (Smith, 2013; McDaniel et al., 1989), supporting the present hypothesis of contextual facilitation.

Overall, the purpose of this research was to assess how context reinstatement influences verbal working memory performance. The first three experiments assess *context-dependent working memory*, without any induction of proactive interference. The last two experiments additionally include a recency-based proactive interference induction to evaluate if context reinstatement affects familiarity-based proactive interference within verbal working memory.

## 2 Experiment 1

### 2.1 Method

#### 2.1.1 Participants

A total of 50 participants received course credit for their participation. This sample size was selected to be similar to sample sizes that have examined context-dependent memory effects (e.g., Murnane and Phelps, 1994; Murnane and Phelps, 1995), to ensure that they would be large enough to detect effect sizes of a similar magnitude. No exclusions were necessary for the working memory analysis, *n* = 50 (25 women, 24 men, 1 non-binary; *M* = 19.32 years; *SE* = 0.17). One participant was excluded from the long-term memory analysis for failure to follow task instructions.

This study was approved by the institutional review board (IRB) at the University of Tampa. All participants provided written informed consent and were treated within the ethical guidelines of the American Psychological Association (APA) and the Declaration of Helsinki.

#### 2.1.2 Materials

Verbal stimuli were drawn from the MRC Psycholinguistic Database,^1^ and all met the following characteristics: 3–7 letters in length, 1–3 syllables, Kucera-Francis written frequency of 10–150, familiarity ratings of 400–700, and concreteness ratings of 300–600. All color words (e.g., “blue,” “gold”) were excluded to avoid Stroop-like effects (Stroop, 1935). Highly emotional words (e.g., “assault,” “murder”) were also excluded, as emotional content has been shown to influence memory and proactive interference (e.g., Levens and Phelps, 2008; LaBar and Phelps, 1998).

#### 2.1.3 Procedure

##### 2.1.3.1 Working memory phase

**Figure 1:**
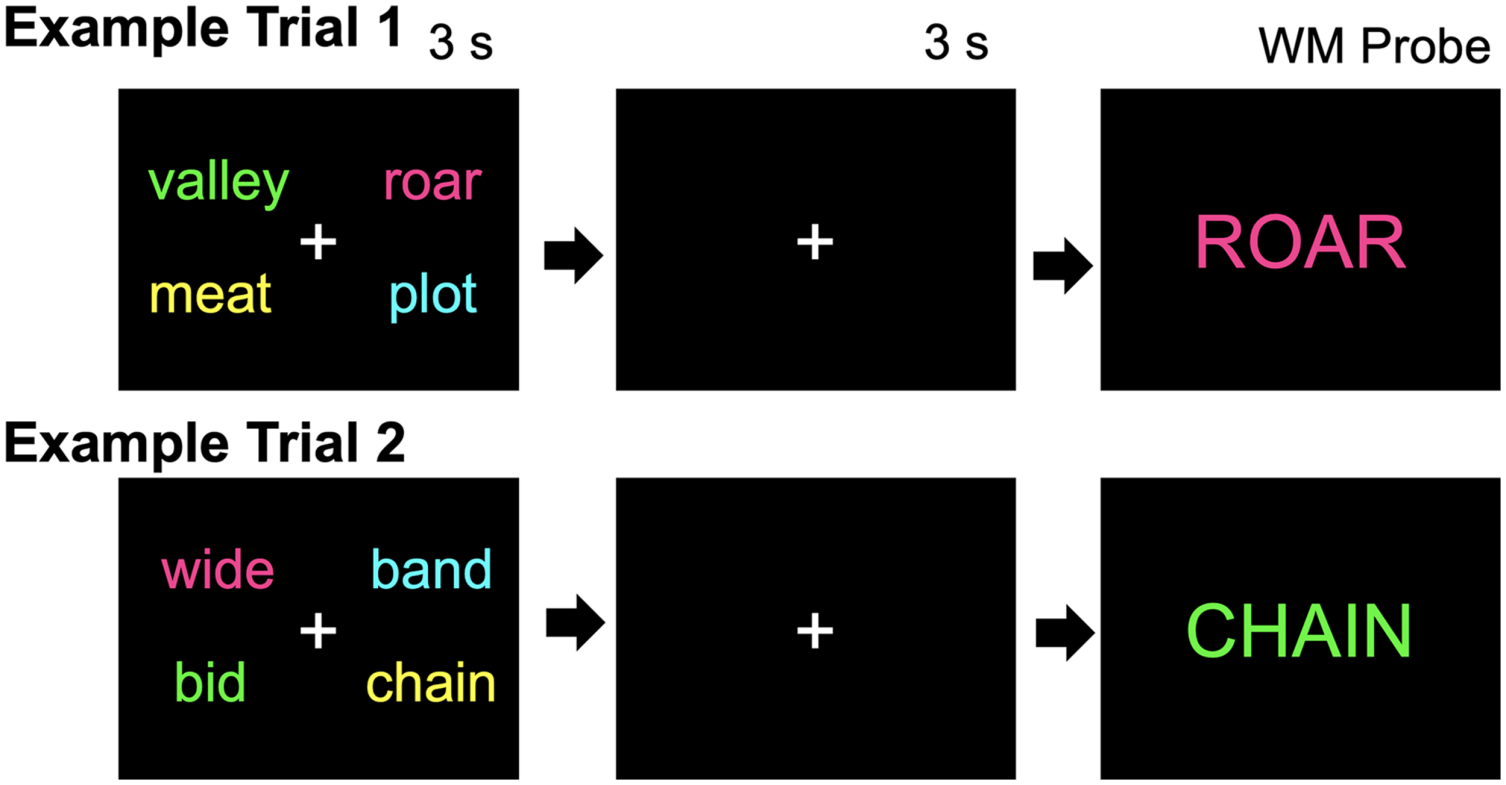
Two example working memory trials in Experiment 1. On each trial, 4 words are studied. After a short delay of 3 s, a single probe word is shown. The participant must click to indicate whether or not the single word was studied on that trial, as quickly and accurately as possible. In Example Trial 1, the single word “ROAR” was presented in the same font color in which it was studied (a Match probe), whereas in Example Trial 2, the single word “CHAIN” was presented in a different font color (a Mismatch probe). Note that in Experiment 1 the position of the font colors was random, whereas in all other experiments the color position was fixed.

The experiment implemented a verbal working memory item-recognition task programed in E-Prime 3.0 (Psychology Software Tools). Participants studied 4 words presented simultaneously on a computer screen for 3 s (e.g., cross, vacuum, jump, autumn). Two words were presented to the left and to the right of the fixation cross. The words disappeared, and after a 3-s retention interval delay, a single probe word (e.g., AUTUMN) was shown in the center of the screen (maximum presentation time = 3 s; termination upon response; inter-trial interval (ITI) = 1.5 s). Participants indicated whether or not the probe word was a word that they were supposed to remember on that trial by pressing a corresponding mouse button (Left = “Yes”; Right = “No”). Importantly, encoding context was manipulated by altering font color. Participants studied words in different colors, and, after the delay, the probe word was presented in the studied font color (Match probes) or in a different font color (Mismatch probes). Participants were instructed that only the word itself needed to be included in the set of studied words, regardless of which color it was presented on the screen—color was irrelevant. Accuracy and response times to these working memory probes were measured. Only correct responses are included in the response time analyses. Further, any responses faster than 200 milliseconds were excluded (e.g., Whelan, 2008), as these responses are too fast to be true memory decisions (e.g., Whelan, 2008; Festini, 2020). In Experiment 1, font color location was not fixed. For example, the ink color magenta could have been presented in any corner of the screen and varied from trial to trial. See Figure 1 for a task diagram.

Participants completed a total of 56 working memory trials, amounting to 224 total words studied in the working memory phase. The encoded stimuli were balanced such that the contextual font color appeared equally often in each of the 4 possible screen positions. The response rate was set at 50% “Yes” and 50% “No” responses for perfect performance, such that 28 trials included probes that were studied (requiring a “Yes” response) and 28 trials included probes that were unstudied (New probes, requiring a “No” response). Of the 28 “Yes” trials, 14 were Match probes and 14 were Mismatch probes,^2^ counterbalanced such that the same probe word appeared equally often as either a Match or Mismatch probe between participants. Participants should say “Yes” to both Match and Mismatch probes, as they were both included in the set of words they were instructed to remember on that trial. The Match probes reinstate the encoding context whereas the Mismatch probes do not. Moreover, probe words originated equally often from the right or left side of the computer screen, appeared equally often in each of the 4 possible screen positions, appeared equally often in each font color, and appeared equally often in each color in each of the screen positions. All trials were presented in random order.

##### 2.1.3.2 Long-term memory phase

A surprise long-term memory (LTM) assessment was given after the entire working memory phase was complete. In the LTM phase, participants were shown a single probe word one-at-a-time in the center of the screen (maximum presentation of LTM probe word = 4 s; termination upon response to probe; ITI = 1.75 s), and their task was to indicate with a mouse button press whether or not the single word was studied at any time during the prior working memory phase. Participants were instructed to make their responses as quickly and as accurately as possible.

The 56 LTM trials mirrored the probe types and probe rate of the working memory phase, such that there were 14 LTM Match probes, 14 LTM Mismatch probes, and 28 LTM New probes, amounting to a 50% “Yes” and 50% “No” response rate. LTM probes were also balanced such that they appeared equally often in each of the possible font colors and had previously appeared in each of the possible screen positions. Importantly, a word that was probed in the working memory phase could not be probed again in the long-term memory phase. All LTM trials were randomized.

### 2.2 Results

#### 2.2.1 Working memory

**Figure 2:**
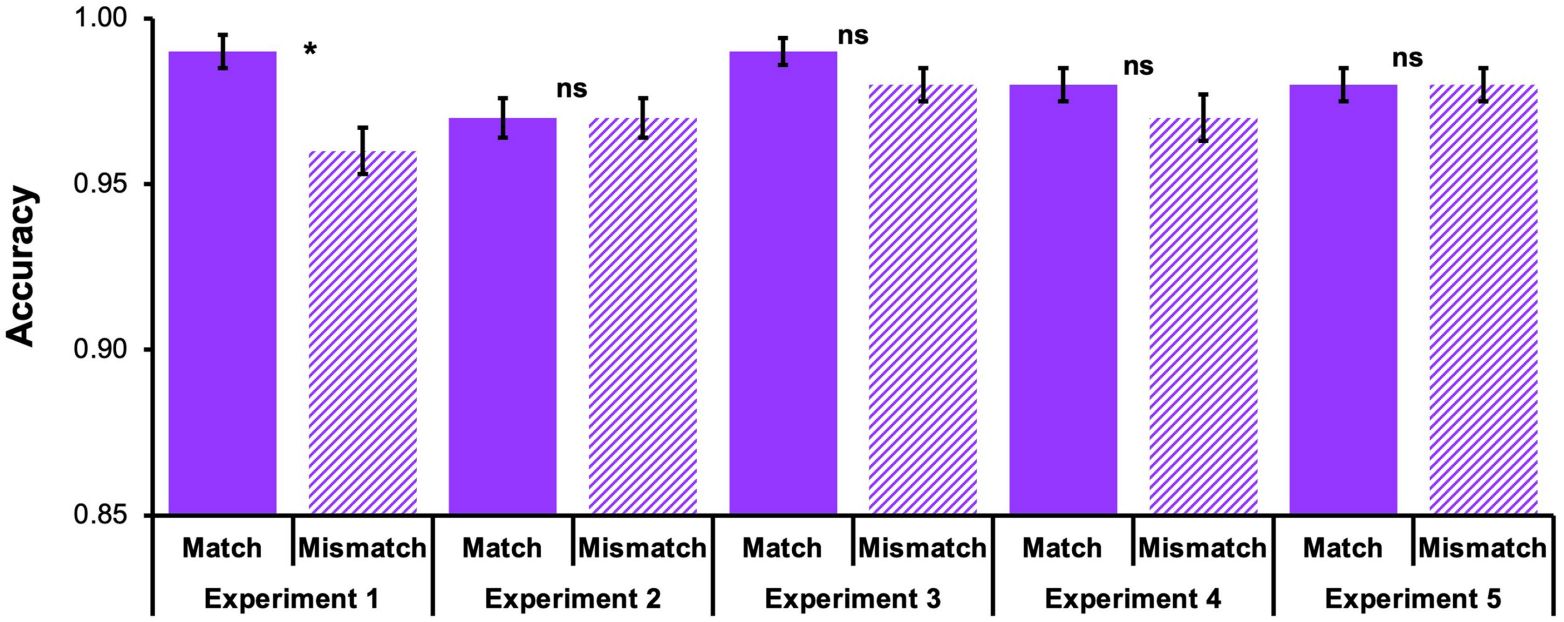
Working memory accuracy (% correct) for Match probes and Mismatch probes across all 5 experiments. Mean accuracy (±1 standard error) for working memory item recognition probes. Most comparisons were non-significant, *ps* > 0.05. Only Experiment 1 showed a significant difference, *p* < 0.05.

**Figure 3:**
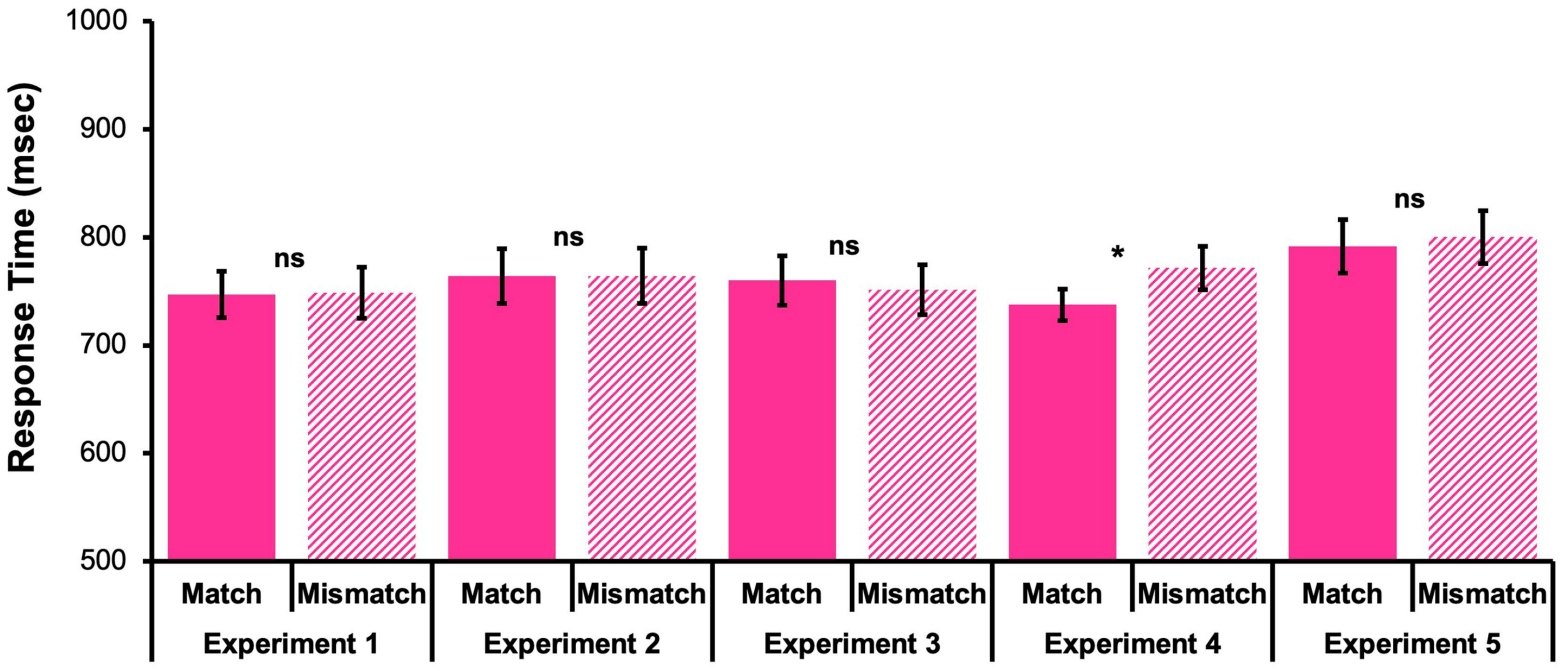
Response times (RTs) within working memory for Match probes and Mismatch probes across all 5 experiments. Mean (RTs ± 1 standard error) for working memory item recognition probes. Most comparisons were non-significant, *ps* > 0.05. Only Experiment 4 showed a significant difference, *p* < 0.05.

A Shapiro–Wilk test confirmed that the accuracy data deviated from a normal distribution, *p* < 0.001. Consequently, accuracy performance was evaluated with non-parametric tests which do not require an assumption of normality. Assessment of the question-of-interest revealed that participants had significantly better accuracy for Match probes than Mismatch probes, *Z* = −3.01, *p* = 0.003 (see Figure 2). Next, to evaluate response times,^3^ a Wilcoxon Signed Ranks Test revealed that response times to Match probes and Mismatch probes were not significantly different, *Z* = −0.34, *p* = 0.732 (see Figure 3). See Table 1 for descriptive statistics.

**Table 1:** Mean accuracy and mean response time (RT) as a function of probe type in working memory for each experiment, with standard error reported in parentheses.

Measure	Experiment	Match	Mismatch	New
Accuracy	1*	0.99 (0.005)	0.96 (0.007)	0.99 (0.002)
	2	0.97 (0.006)	0.97 (0.006)	0.99 (0.002)
	3	0.99 (0.004)	0.98 (0.005)	0.99 (0.003)
	4	0.98 (0.005)	0.97 (0.007)	1.00 (0.003)
	5	0.98 (0.005)	0.98 (0.005)	1.00 (0.002)
RT	1	747.02 (21.56)	748.81 (23.61)	759.74 (25.85)
	2	764.24 (25.51)	764.22 (25.55)	771.10 (22.99)
	3	760.04 (23.01)	751.58 (23.29)	755.28 (25.59)
	4*	737.58 (14.68)	771.69 (20.11)	732.32 (17.73)
	5	791.59 (24.93)	800.36 (24.48)	764.68 (22.80)

Mean RTs are reported in milliseconds, and only correct responses are included in the RT average. Note that in the working memory phase Match and Mismatch probes required a “Yes” response, whereas New probes required a “No” response.

Asterisks (*) indicate significant differences between Match and Mismatch conditions, *p* < 0.05. All other comparisons were non-significant. See text for more details.

#### 2.2.2 Long-term memory

**Figure 4:**
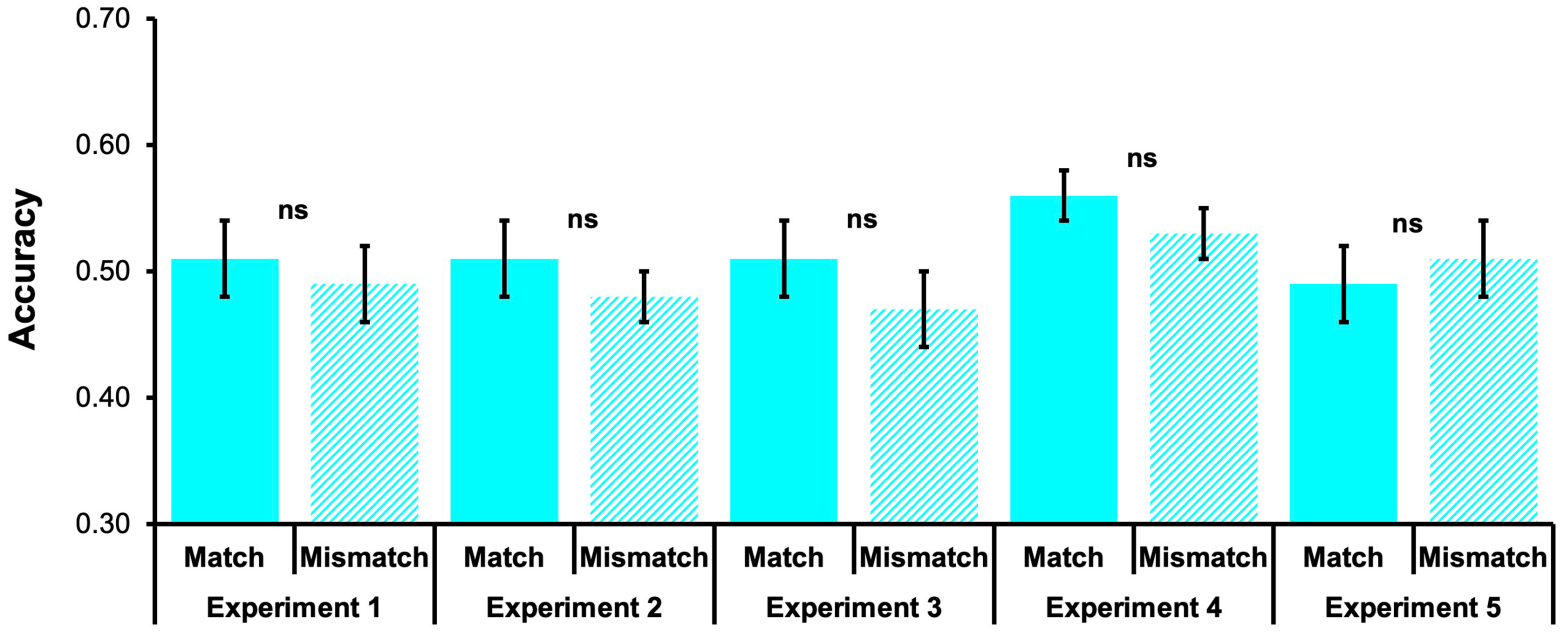
Long-term memory accuracy (% correct) for Match probes and Mismatch probes across all 5 experiments. Mean accuracy (±1 standard error) for incidental long-term memory recognition probes. All comparisons were non-significant, *ps* > 0.05.

There was no significant difference in long-term memory accuracy for probes that were presented in the same (Match) versus a different (Mismatch) font color as during encoding, *t*(48) = 0.67, *p* = 0.504, Cohen’s *d* = 0.10, 95% CI [−0.19, 0.38] (see Figure 4; Table 2).

**Table 2:** Incidental long-term memory mean accuracy as a function of probe type for each experiment, with standard error reported in parentheses.

Measure	Experiment	Match	Mismatch	New
Accuracy	1	0.51 (0.03)	0.49 (0.03)	0.70 (0.03)
	2	0.51 (0.03)	0.48 (0.02)	0.73 (0.02)
	3	0.51 (0.03)	0.47 (0.03)	0.72 (0.02)
	4	0.56 (0.02)	0.53 (0.02)	0.79 (0.02)
	5	0.49 (0.03)	0.51 (0.03)	0.82 (0.02)

Note that in the long-term memory phase Match and Mismatch probes required a “Yes” response, whereas New probes required a “No” response.

All long-term memory comparisons between Match and Mismatch conditions were non-significant.

### 2.3 Discussion

The results of Experiment 1 provide mixed evidence for and against context-dependent working memory. Only working memory accuracy was significantly superior for Match probes with a reinstated context. Neither working memory response times nor surprise long-term memory assessments indicated any difference in performance when font color context was reinstated or not.

One possibility is that allowing the font color to be presented in varying screen locations from trial to trial may have weakened the effect of context. To evaluate this possibility, in Experiment 2 the font color was always presented in the same location on each trial. Stable font color location may induce a stronger color context, as participants can predict which font color will appear in which screen location and become more accustomed to the color context.

## 3 Experiment 2

### 3.1 Method

#### 3.1.1 Participants

A total of 52 participants received course credit for their participation. This sample size was selected to be consistent with the sample size of Experiment 1. After exclusions, a final sample of *n* = 50 (34 women, 16 men, 0 non-binary) were included in the analyses (*M* = 19.66 years; *SE* = 0.29). One participant was excluded for poor working memory performance that fell 2.5 standard deviations below the mean, and one participant was excluded due to a computer malfunction. For the long-term memory analyses, one participant was excluded for poor long-term memory performance that fell 2.5 standard deviations below the mean.

#### 3.1.2 Materials and procedure

The procedure was identical for Experiment 2, with the exception that font color was now *always* presented in the identical screen location on each trial. Specifically, words were always presented in one of two possible stable color positions, between-participants: (1) magenta font color in the top-left corner, cyan font color in the top-right corner, yellow font color in the bottom-left corner, and lime font color in the bottom-right corner or (2) cyan font color in the top-left corner, lime font color in the top-right corner, magenta font color in the bottom-left corner, and yellow font color in the bottom-right corner. Two possible stable color positions were included to ensure that one particular color ordering was not driving the results.

### 3.2 Results

#### 3.2.1 Working memory

Participants exhibited similar accuracy regardless of whether a studied probe matched or did not match the encoded font color,^4^ *Z* = −0.49, *p* = 0.621 (see Figure 2). Evaluation of response times with a paired samples *t*-test^5^ indicated no difference for Match probes and Mismatch probes, *t*(49) = 0.002, *p* = 0.998, Cohen’s *d* = 0.00, 95% CI [−0.28, 0.28] (see Figure 3; Table 1).

#### 1.1.1 Long-term memory

There was no significant difference in long-term memory accuracy for probes that were presented in the same (Match) versus a different (Mismatch) font color as during encoding, *t*(48) = 1.09, *p* = 0.279, Cohen’s *d* = 0.16, 95% CI [−0.13, 0.44] (see Figure 4; Table 2).

### 3.3 Discussion

Experiment 2 did not find any evidence in support of context-dependent working memory facilitation. Neither working memory accuracy nor response times were influenced by context reinstatement. Incidental long-term memory was also unaffected by reinstating the encoded font color.

Despite font color being presented in a stable screen location, there was no evidence for context-dependent facilitation within working memory. In Experiment 3, background color was manipulated, instead of font color, to provide larger swaths of color to encode and a more salient context. All words were written in black font atop a colorful rectangle (cf. Dulsky, 1935; Weiss and Margolius, 1954). These background colors were presented in stable screen locations in an attempt to maximize color context stability and saliency.

## 4 Experiment 3

### 4.1 Method

#### 4.1.1 Participants

A total of 55 participants received course credit for their participation. This sample size was selected to be consistent with the sample size of the prior experiments. After exclusions, a final sample of *n* = 51 (43 women, 8 men, 0 non-binary) were included in the analyses (*M* = 18.94 years; *SE* = 0.15). Four participants were excluded for poor working memory performance that fell 2.5 standard deviations below the mean.

#### 4.1.2 Materials and procedure

**Figure 5:**
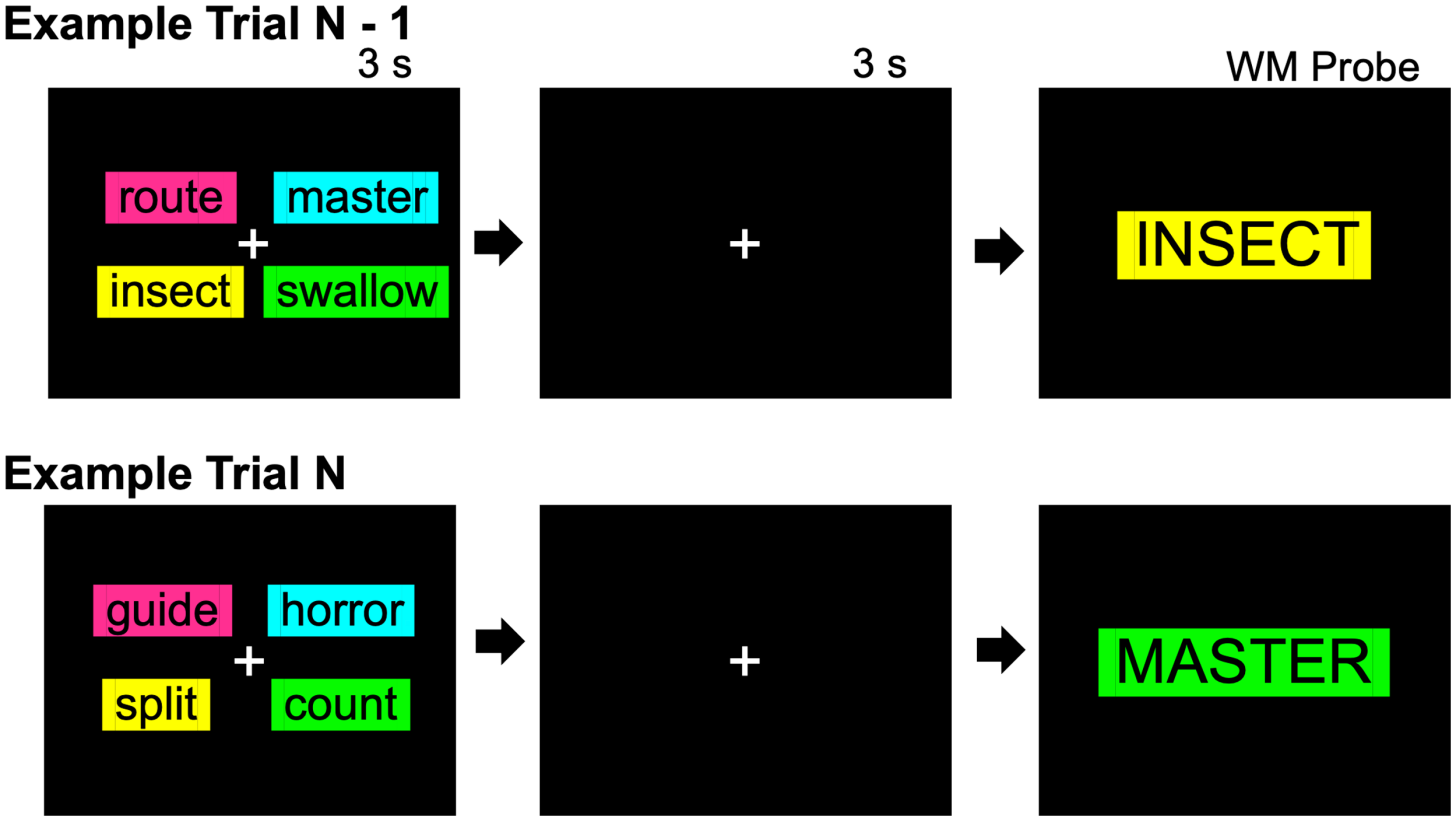
Two example working memory trials from Experiment 5 with a recent probes manipulation to induce proactive interference. On each trial, 4 words are studied. After a short delay of 3 s, a single probe word is shown. The participant must click to indicate whether or not the single word was studied on that trial, as quickly and accurately as possible. In Example Trial N − 1, the single word “INSECT” was presented in the same background color in which it was studied (a Match probe), whereas in Example Trial N, the single word “MASTER” was presented on the *prior* trial (trial N − 1) in a different background color (a Mismatch-Recent probe). Only Experiments 4 and 5 induced proactive interference with this recent probes manipulation. Experiment 4 used font color as the encoding context, whereas Experiment 5 used background color as the encoding context (depicted).

The procedure for Experiment 3 was identical to Experiment 2, with the exception that *background color* was now manipulated instead of font color. Words were displayed in black font on top of colorful backgrounds. Color context was stable across all trials, in one of two possible stable configurations, between-participants: (1) magenta background rectangles were always presented in the top-left corner of the screen, cyan rectangles were always presented in the top-right corner, yellow rectangles were always presented in the bottom-left, and lime rectangles were always presented in the bottom-right or (2) cyan background rectangles were always presented in the top-left corner of the screen, lime rectangles were always presented in the top-right corner, magenta rectangles were always presented in the bottom-left, and yellow rectangles were always presented in the bottom-right. See Figure 5 for a general depiction of the presentation of stable background color (although the recent probes manipulation within the figure is only relevant to Experiments 4 and 5).

### 4.2 Results

#### 4.2.1 Working memory

Participants exhibited similar accuracy regardless of whether a studied probe matched or did not match the encoded font color,^6^ *Z* = −0.88, *p* = 0.378 (see Figure 2). Next, a Wilcoxon Signed Ranks Test^7^ indicated that response times to Match probes and Mismatch probes were not significantly different, *Z* = −0.262, *p* = 0.793 (see Figure 3; Table 1).

#### 4.2.2 Long-term memory

There was no significant difference in long-term memory accuracy for probes that were presented in the same (Match) versus a different (Mismatch) font color as during encoding, *t*(50) = 1.34, *p* = 0.188, Cohen’s *d* = 0.19, 95% CI [−0.09, 0.46] (see Figure 4; Table 2).

### 4.3 Discussion

Experiment 3 did not find any evidence in support of context-dependent working memory, even when background color was used as the encoding context. Neither working memory accuracy nor response times were influenced by background color reinstatement. Incidental long-term memory performance was also not influenced by reinstatement of the encoded background color context.

To examine whether indirect measures of memory strength would have more success in revealing encoding specificity effects within working memory, Experiments 4 and 5 additionally induced recency-based proactive interference. Induction of proactive interference can provide an additional window into the extent of working memory context reinstatement effects, as stronger working memory representations have been shown to result in larger proactive interference (e.g., Festini, 2020; Festini and Reuter-Lorenz, 2014; Jantz et al., 2020). If working memory context reinstatement strengthens recognition memory, Match-Recent probes should exhibit significantly more proactive interference than Mismatch-Recent probes.

## 5 Experiment 4

One prior study has examined whether recency-based proactive interference is influenced by matching study/test perceptual characteristics. Craig et al. (2013) manipulated whether recent probes had identical color and font attributes (font type, boldness, italics) or opposite attributes. Importantly, they observed no difference in recency-based proactive interference for format-repeat versus format-change recent probes. Hence there is precedent for a lack of encoding specificity effects on working memory proactive interference. Nevertheless, it is worthwhile to empirically evaluate whether this effect replicates, as the prior findings may have been underpowered (18 participants only) and included a different manipulation of context.

### 5.1 Method

#### 5.1.1 Participants

A total of 60 participants received course credit for their participation. This sample size was selected to be consistent with the sample size of the prior experiments. After exclusions, a final sample of *n* = 56 (50 women, 6 men, 0 non-binary) were included in the analyses (*M* = 18.54 years; *SE* = 0.13). Two participants were excluded for failure to follow task instructions. Two additional participants were excluded for poor working memory performance that fell 2.5 standard deviations below the mean. In the LTM analysis, one participant was excluded for failure to follow task instructions.

#### 5.1.2 Materials and procedure

Experiment 4 followed an identical procedure to Experiment 2, except proactive interference was also induced with a recent probes manipulation, also known as a recent-negatives or recency-probes task (e.g., Atkins et al., 2011; Badre and Wagner, 2005; D’Esposito et al., 1999; Festini and Reuter-Lorenz, 2014; Jonides and Nee, 2006; Mercer, 2025; Monsell, 1978). Context was manipulated with font color presented in one of two possible stable screen locations across all trials, between-participants (same as in Experiment 2).

To enable the proactive interference induction, an additional 4 trials were added to ensure balancing of all probe types, amount to a total of 60 working memory trials. The response rate was consistent at 50% “Yes” (30 trials) and 50% “No” (30 trials). As in the prior experiments, half of the probes that required a “Yes” response were presented in the same font color as studied (Match; 15 trials) and half were presented in a different font color (Mismatch; 15 trials), counterbalanced such that the same probe word was presented equally often as a Match or Mismatch probe between-participants.

The proactive interference induction introduced two additional probe types: Match-Recent (10 trials) and Mismatch-Recent probes (10 trials). A Match-Recent probe presents a word that was studied on the *prior* trial as opposed to the current trial and was presented in the studied font color. Respectively, a Mismatch-Recent probe presents a word that was studied on the *prior* trial and was presented in a different font color. Both of these probe types require a “No” response because they do not satisfy the requirement of being a single word that was studied on the current trial. The remaining “No” probes (10 trials) consisted of unstudied New probes, as in the prior experiments. All trials were pseudorandomized in a fixed order to permit induction of proactive interference and the following criteria: Probe words appeared equally often from the right and left side of the computer screen and approximately equally often in each color and in each of the 4 possible screen positions. No more than 3 of the same responses were warranted in a row, and no more than 2 of the same probe type were presented in a row. Importantly, Recent probe words appeared equally often as a Match-Recent or Mismatch-Recent probe, between-participants, and originated approximately equally often from each side of the screen and from each of the possible screen positions. See Figure 5 for a task diagram (except Experiment 4 utilized font color as opposed to background color).

The long-term memory phase paralleled the prior experiments, with the exception of 4 additional trials to correspond to the working memory phase. There were 15 LTM-Match probes, 15 LTM-Mismatch probes, and 30 LTM-New probes. Proactive interference was not induced in long-term memory. The response rate was consistent at 50% “Yes” and 50% “No.” Long-term memory probes were balanced to appear equally often in each color, approximately equally often in each list position, and to originate equally often from the right and left sides of the screen. The same word was never probed in both the WM and LTM phases.

### 5.2 Results

#### 5.2.1 Working memory accuracy

Examination of the effect of context reinstatement on accuracy for studied probes revealed similar accuracy regardless of whether a studied probe matched or did not match the encoded font color,^8^ *Z* = −1.26, *p* = 0.209 (see Figure 2).

A Friedman Test indicated significant differences in accuracy, among probe types that required a “No” response, *χ*^2^(2) = 11.18, *p* = 0.004. Follow-up Wilcoxon Signed Ranks Tests indicated significant proactive interference within accuracy performance for both recent probe types, such that participants were less accurate for Match-Recent probes than New probes, *Z* = −2.86, *p* = 0.004, and less accurate for Mismatch-Recent probes than New probes, *Z* = −3.09, *p* = 0.002. Yet, there were no significant differences in accuracy for Match-Recent and Mismatch-Recent probes, *Z* = −0.16, *p* = 0.872 (see Table 3).

**Table 3:** Mean accuracy and mean response time (RT) for proactive interference-inducing recent probes and new probes in working memory, with standard error reported in parentheses.

Measure	Experiment	Match-Recent	Mismatch-Recent	New
Accuracy	4	0.97 (0.009)	0.97 (0.008)	1.00 (0.003)
	5	0.98 (0.006)	0.98 (0.005)	1.00 (0.002)
RT	4	866.09 (24.21)	849.58 (22.98)	732.32 (17.73)
	5	908.52 (26.67)	888.66 (24.26)	764.68 (22.80)

Mean RTs are reported in milliseconds, and only correct responses are included in the RT average. All probes required a “No” response.

All comparisons between Match-Recent and Mismatch-Recent conditions were non-significant. All comparisons between recent probes and new probes revealed significant proactive interference, all *ps* < 0.012.

#### 5.2.2 Working memory response time

**Figure 6:**
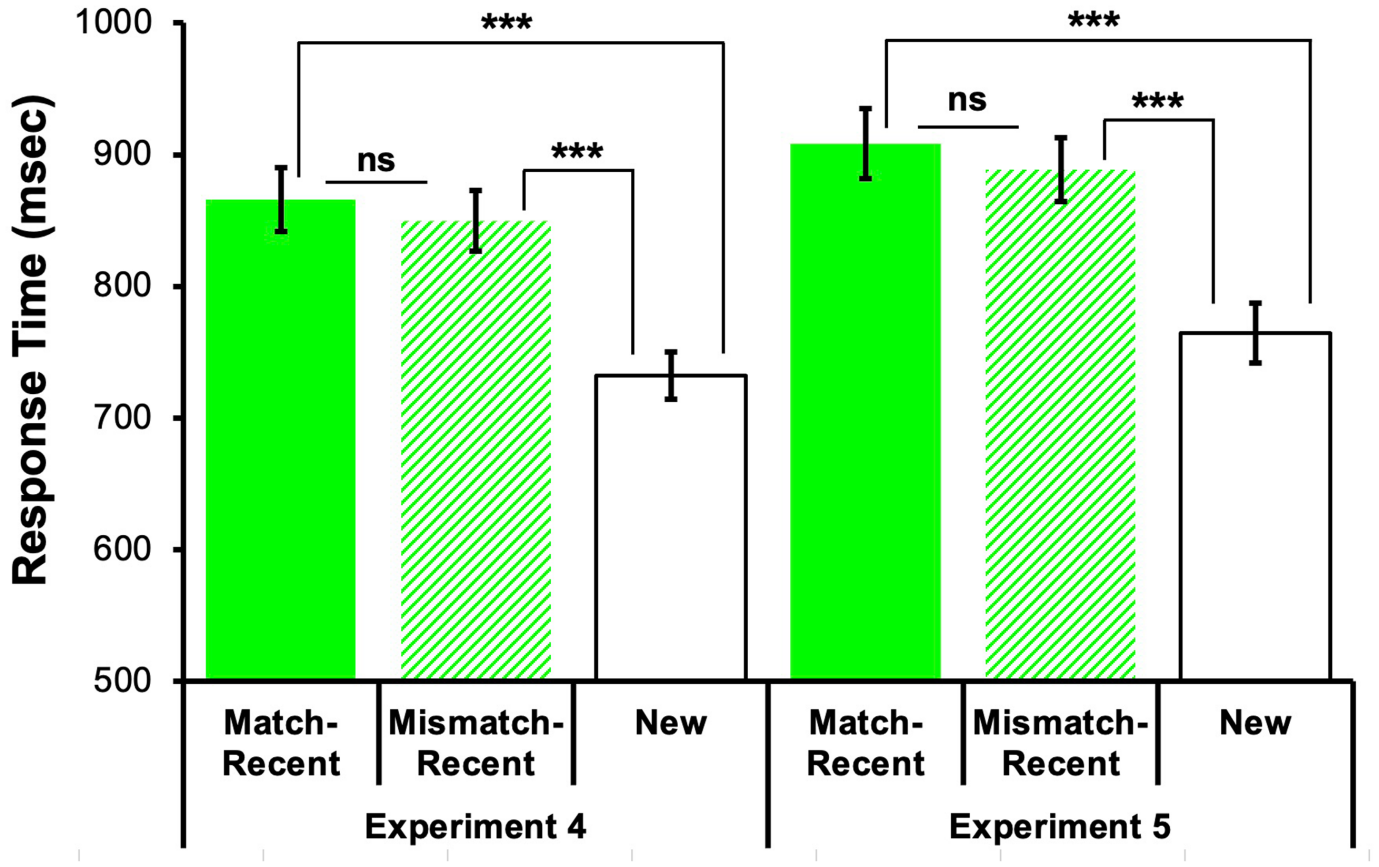
Similar proactive interference for Match-Recent and Mismatch-Recent probes in Experiments 4 and 5. Mean response times (RTs ± 1 standard error) for working memory item recognition probes. Consistent results across Experiments 4 (font color context) and 5 (background color context): Significant proactive interference was observed (i.e., longer RTs) for both Match-Recent and Mismatch-Recent probes relative to New (control) probes (*ps* < 0.001, ***), although context reinstatement did *not* influence recency-based proactive interference within working memory (i.e., no significant differences in response times between Match-Recent and Mismatch-Recent probes, *ps* > 0.1, not significant, ns).

Due to the differences in the decision being made, probe types that required a “No” and “Yes” response were analyzed separately. A repeated measures ANOVA^9^ compared average response times for all 3 negative probe types (New, Match-Recent, and Mismatch-Recent). There were significant differences in response times, *F*(2, 110) = 48.55, *p* < 0.001, *partial η*^2^ = 0.47. Follow-up Bonferroni-corrected pairwise comparisons revealed that participants took significantly longer to reject Match-Recent and Mismatch-Recent probes than New probes, both *ps* < 0.001, indicative of significant proactive interference for both recent probe types. Nevertheless, there was no significant difference between response time to correctly reject Match-Recent and Mismatch-Recent probes, *p* = 0.989 (see Figure 6; Table 3).

Proactive interference scores were also calculated for each participant by subtracting the average time to correctly reject a New probe from the average time to correctly reject a Recent probe, resulting in the average additional processing time required to overcome proactive interference.^10^ A paired samples *t*-test revealed no significant difference in proactive interference for Match-Recent (*M* = 133.77, *SE* = 12.93) versus Mismatch-Recent probes (*M* = 117.26, *SE* = 14.43), *t*(55) = 0.98, *p* = 0.330, Cohen’s *d* = 0.13, 95% CI [−0.13, 0.39]. Therefore, although there were significant amounts of proactive interference for both recent probe types (i.e., over 115 milliseconds of additional processing time to overcome the familiarity of the recent probe), it did not matter whether the recent probe had the color context reinstated or not.

Finally, assessments of response times to “Yes” trials indicated that participants were significantly quicker to respond to studied Match probes than studied Mismatch probes, *t*(55) = −3.00, *p* = 0.004, Cohen’s *d* = −0.40, 95% CI [−0.67, −0.13] (see Figure 3; Table 1).

#### 5.2.3 Long-term memory performance

There was no significant difference in long-term memory accuracy for probes that were presented in the same (Match) versus a different (Mismatch) font color as during encoding, *t*(54) = 1.19, *p* = 0.238, Cohen’s *d* = 0.16, 95% CI [−0.11, 0.43] (see Figure 4; Table 2).

### 5.3 Discussion

Experiment 4 included an induction of recency-based proactive interference. Participants exhibited significant levels of proactive interference, evident in both accuracy and response time data. Familiar, recent probes resulted in more errors and longer response times than new, unstudied probes (the control condition). Yet, context reinstatement did not influence the magnitude of proactive interference. There were no differences in accuracy nor response times to recent probes regardless of whether or not font color context was reinstated.

For studied memoranda (for which proactive interference was not induced), Experiment 4 observed that neither working memory accuracy nor incidental long-term memory accuracy were influenced by context reinstatement. Nevertheless, participants were significantly faster to respond to studied Match probes than studied Mismatch probes.

To evaluate if larger swaths of background color would provide more salient color context to reveal context reinstatement effects, Experiment 5 manipulated proactive interference and presented word memoranda atop colorful background rectangles that were always presented in the same screen position.

## 6 Experiment 5

### 6.1 Method

#### 6.1.1 Participants

A total of 61 participants received course credit for their participation. This sample size was selected to be consistent with the sample size of the prior experiments. After exclusions, a final sample of *n* = 58 (40 women, 17 men, 1 non-binary) were included in the analyses (*M* = 18.90 years; *SE* = 0.14). Three participants were excluded for poor working memory performance that fell 2.5 standard deviations below the mean.

#### 6.1.2 Materials and procedure

Experiment 5 was identical to Experiment 4, with the exception that background color was manipulated as opposed to font color. Background colors were presented in stable locations across all trials and proactive interference was also induced (see Figure 5).

### 6.2 Results

#### 6.2.1 Working memory accuracy

Examination of the effect of context reinstatement on accuracy for studied probes revealed similar accuracy regardless of whether a studied probe matched or did not match the encoded font color,^11^ *Z* = −0.80, *p* = 0.424 (see Figure 2).

A Friedman Test indicated significant differences in accuracy, among probe types that required a “No” response, *χ*^2^(2) = 11.63, *p* = 0.003. Follow-up Wilcoxon Signed Ranks Tests indicated significant proactive interference for both recent probe types, such that participants were less accurate for Match-Recent probes than New probes, *Z* = −2.53, *p* = 0.011, and less accurate for Mismatch-Recent probes than New probes, *Z* = −3.32, *p* < 0.001. Yet, there were no significant differences in accuracy for Match-Recent and Mismatch-Recent probes, *Z* = −0.73, *p* = 0.467 (see Table 3).

#### 6.2.2 Working memory response time

A Friedman’s test^12^ indicated that there were significant differences in response times for the 3 negative probe types, *χ*^2^(2) = 58.17, *p* < 0.001. Follow-up Wilcoxon tests revealed that participants took significantly longer to reject Match-Recent and Mismatch-Recent probes than New probes, both *ps* < 0.001, indicative of significant proactive interference. Nevertheless, there was no significant difference between response times to correctly reject Match-Recent and Mismatch-Recent probes, *p* = 0.108 (see Figure 6; Table 3).

A comparable Friedman’s test on proactive interference scores themselves revealed no significant difference in proactive interference for Match-Recent (*M* = 143.84, *SE* = 12.20) versus Mismatch-Recent probes (*M* = 123.98, *SE* = 14.00), *Z* = −1.61, *p* = 0.108. Therefore, although there were significant amounts of proactive interference for both recent probe types (i.e., over 120 milliseconds of additional processing time to overcome the familiarity of the recent probe), it did not matter whether the recent probe had the color context reinstated or not.

Finally, a Wilcoxon test of response times to “Yes” trials indicated no significant difference in response times to studied Match probes and studied Mismatch probes, *Z* = −0.83, *p* = 0.407 (see Figure 3; Table 1).

#### 6.2.3 Long-term memory performance

There was no significant difference in long-term memory accuracy for probes that were presented in the same versus a different font color as during encoding, *t*(57) = −1.30, *p* = 0.199, Cohen’s *d* = −0.17, 95% CI [−0.43, 0.09] (see Figure 4; Table 2).

### 6.3 Discussion

Experiment 5 largely replicated the effects of Experiment 4. Significant levels of proactive interference were observed, such that participants were less accurate and slower to correctly respond to recent probes. However, proactive interference levels within working memory were unimpacted by background color context reinstatement.

Studied probes were also uninfluenced by context reinstatement. In Experiment 5, there were no significant differences in working memory accuracy, working memory response times, nor incidental long-term memory accuracy for studied words presented on the same or different background color contexts. Therefore, there is no consistent evidence that simultaneous induction of proactive interference magnifies working memory context reinstatement effects. Rather, the majority of evidence across all experiments finds no performance differences regardless of whether or not working memory encoding color context is reinstated.

## 7 General discussion

Encoding specificity within verbal working memory was evaluated in a series of five experiments. All experiments implemented a Sternberg item-recognition verbal working memory task (e.g., Sternberg, 1966) at a set size of 4, in which a single recognition probe was presented to participants to be recognized. This enabled the color context to be reinstated (i.e., match) or altered (i.e., mismatch). Experiment 1 manipulated font color context, where different font colors could appear in each of the four screen locations. Experiment 2 held the font color location constant in an attempt to increase context stability. Experiment 3 used background colors presented at stable screen locations to attempt to increase color context saliency. The last two experiments also induced proactive interference by occasionally presenting recent probes that originated from the *prior* trial as opposed to the current trial, with stable font color (Experiment 4) and stable background color (Experiment 5).

Across all experiments, most evidence failed to find support for verbal memory facilitation with color context reinstatement in working memory. That is, across 19 assessments (with only two exceptions), participants exhibited equivalent response times and equivalent accuracies for verbal working memory recognition probes that reinstated the same color context (i.e., Match probes) as those that had a different color context (i.e., Mismatch probes). Moreover, similar levels of proactive interference were evident for recent probes that were presented in a matching or mismatching color context, consistent with Craig et al. (2013). Subsequent incidental long-term memory assessments also found no evidence of context-dependent memory for items encoded during a working memory task. Overall, consistent support for context-dependent memory effects within verbal working memory was absent.

The absence of context reinstatement effects may be attributed by some to the nature of the recognition assessment, as long-term memory assessments of context-dependent memory can fail to observe reinstatement effects with recognition memory tests (e.g., Eich, 1985; Jacoby, 1983; Smith et al., 1978; Godden and Baddeley, 1980). Baddeley (1982) posited that context reinstatement would *not* impact recognition memory unless participants were directed to process both memoranda and contextual features interactively (cf. Kirmsse et al., 2022). Smith et al. (1978) proposed that more difficult recall tests are more likely to encourage use of environmental context retrieval cues, whereas in recognition tests the item itself is presented, which reduces reliance on other contextual cues (see also Eich, 1980; Geiselman and Bjork, 1980; Spear, 1978). Nevertheless, context-dependent memory effects *have* been observed in some recognition tests (e.g., Dalton, 1993; Krafka and Penrod, 1985; Smith, 1986; Smith and Vela, 1992; Isarida et al., 2005; Hayes et al., 2007). Therefore, although the nature of the recognition assessment likely contributed to the absent/weak effects, reinstatement effects are not unheard of in recognition assessments.

Some may also argue that the absence of a context-reinstatement effect within the present incidental long-term memory assessment is an indication that the context manipulation was flawed. Yet, this is not necessarily true. First, incidental long-term memory performance following encoding during a working memory task has frequently been observed to be weak, due to the surprise nature of the long-term memory assessment (e.g., Festini and Reuter-Lorenz, 2013; Festini and Reuter-Lorenz, 2014; Festini and Reuter-Lorenz, 2017; Festini, 2020; Jantz et al., 2020; Craik et al., 1970). For example, Craik et al. (1970) observed that when immediate free recall was tested, strong long-term memory representations were not needed to be formed for the final items, resulting in weaker subsequent long-term performance (see also Cowan, 2019). Second, prior literature has successfully implemented similar color context manipulations within long-term memory tasks (e.g., Sakai et al., 2010; Murnane and Phelps, 1995; Hockley, 2008), indicating that context reinstatement effects are indeed possible with such color context manipulations.

Nevertheless, it is important for future research to evaluate richer manipulations of environmental contexts within working memory. Even though font manipulations (e.g., Arndt, 2010) and color manipulations have been used with success in prior long-term memory recall (Dulsky, 1935; Weiss and Margolius, 1954; Isarida and Isarida, 2007; Sakai et al., 2010) and recognition tests (Isarida et al., 2005; Hockley, 2008; Murnane and Phelps, 1993; Murnane and Phelps, 1994; Murnane and Phelps, 1995), complex visual contexts have been shown to have stronger effects than background colors (Hockley, 2008). In much research, distinctive environmental context manipulations are frequently used, such as the presence or absence of an audience (Burri, 1931), water versus non-water environments (Godden and Baddeley, 1975), superimposed images or videos (e.g., Hollingworth, 2009; Pan, 1926; Smith and Manzano, 2010), smells (e.g., Aggleton and Waskett, 1999, see Smith and Guthrie, 1921), tastes (e.g., Johnson and Miles, 2007; Johnson and Miles, 2008) and can show stronger effects when the environmental contexts are highly unique rather than more similar (termed cue overload or fan effects, see Smith, 2013, Watkins and Watkins, 1975). Nonetheless, the opposite can also be observed—larger context reinstatement effects when a context spans more rather than fewer items (a global-matching view, see Murnane et al., 1999, Rutherford, 2004, Ensor et al., 2023).

When considering participant strategy, subvocal rehearsal of the working memory items is often proposed as a method to maintain working memory memoranda (e.g., Baddeley, 2012). If participants are implementing this strategy, they are unlikely to also be subvocally rehearsing the font color of the word, as the font color itself is irrelevant to their present goal. Similarly, *overshadowing* has been proposed to occur when one’s limited attention can only sufficiently focus on memoranda themselves, which overshadow the contextual information (Smith, 2013). Nevertheless, item-context binding has also been proposed to occur within working memory (e.g., Lewis-Peacock et al., 2018; Dames and Oberauer, 2022), in which case the context could be expected to influence performance. Future research could increase the relevance of the working memory context, by perhaps instructing participants to occasionally answer a question about the background colors, like an occasional visual short-term memory color wheel probe (cf. Hajonides et al., 2020). If the context becomes relevant, this could potentially heighten the impact of encoding specificity (see Craig et al., 2013; Beaudry et al., 2014). Although one should note that, within the long-term memory literature, the background context is often irrelevant to the prominent memory task, yet context still often substantially influences performance (e.g., Abernethy, 1940; Bilodeau and Schlosberg, 1952; Dulsky, 1935; Hockley, 2008).

Future research could also evaluate whether more complex stimuli, such as background images (cf. Pan, 1926; Smith and Manzano, 2010; Szollosi et al., 2023; Isarida et al., 2021), yield stronger effects. It could be that the present manipulation of font/background color was not a sufficiently strong contextual manipulation. For instance, prior research has indicated that the hippocampus can be recruited during working memory tasks if the memoranda are sufficiently detailed and complex (e.g., Yonelinas, 2013; but see Allen et al., 2014). Font/background color context may not have provided sufficient complexity, despite the observation of effects of font/background color in the long-term memory literature (e.g., Sakai et al., 2010; Murnane and Phelps, 1995; Hockley, 2008).

Given the present (largely null) findings within working memory in comparison to extant evidence of consistent context reinstatement in long-term memory (see Smith, 2013; Smith and Vela, 2001), it is possible that context-dependent memory has a stronger impact on long-term memory than it does on working memory, at least for meaningful verbal memoranda at a set size of 4, as evaluated here. Variations in memorial processes and inherent characteristics of the respective experimental tasks (e.g., including memory strength, delay, set size) may contribute to larger effects of context for long-term memory. For example, in their meta-analysis, Smith and Vela (2001) note that the largest effect sizes were present for studies that implemented the longest delays before memory assessment. In the present working memory paradigm, the delay was only 3 s, which perhaps contributed to the absence of context reinstatement effects and to weaker effects in working memory in general, where delays are, by definition, very short. Furthermore, unlike long-term memory, working memory has limited capacity (e.g., Cowan, 2000), which may similarly attenuate context effects. Because working memory is only capable of maintaining a limited amount of information in a heightened state of accessibility, working memory executive resources may direct most attention to task-relevant information at the expense of contextual information (cf. Zanto and Gazzaley, 2009), more so than is done during long-term memory encoding. That is, as font color was irrelevant to the task response, participants may have strategically guided executive control toward the relevant word meanings and devoted less attention to the font color (cf. Kerzel and Huynh Cong, 2021; Woodman et al., 2012) or ignored this irrelevant feature during the memorial decision (cf. Shin and Ma, 2016). The central executive may have attenuated color context in the episodic buffer in favor of the word features themselves (see Baddeley, 2000), and the focus of attention may have similarly upregulated task-relevant information (see Cowan, 2019; cf. the concept of attribute amnesia, Chen and Wyble, 2015). Future research would benefit from parametrically varying working memory set sizes and manipulating the relevance of contextual information within working memory, to determine the boundary conditions for when context is or is not sufficiently processed within working memory to elicit context reinstatement effects.

Notably, the present study solely evaluated verbal working memory performance at a set size of 4 words. The rationale for this methodological choice was to keep the studied set size within the typical limits of working memory capacity (e.g., Cowan, 2000). Nevertheless, as discussed above, it would be useful for future research to systematically evaluate if the presence/magnitude of context reinstatement effects are influenced by how many items are required to be held in working memory (e.g., 2, 6, 8 items). Using a set size of 4 items also resulted in highly accurate working memory performance, as was expected (cf. e.g., Atkins et al., 2011; Badre and Wagner, 2005; Irlbacher et al., 2014). To address these potential ceiling effects, (a) non-parametric statistical tests were implemented, when needed, that rely on rank ordering and (b) response time data were analyzed that are not vulnerable to ceiling effects. Additionally, all stimuli in the present study were meaningful words. Future work should evaluate if stronger effects of context may be observed for less semantically meaningful stimuli, such as non-words (cf. Mercer, 2025), as the presence of meaning may attract attention and diminish attention toward contextual features. Although the present research provides an initial assessment of context reinstatement, follow-up studies are needed to examine whether context reinstatement effects can be present under certain conditions within verbal working memory.

In the domain of visual working memory/visual short-term memory, there is substantial converging evidence that task-irrelevant features can be dropped quickly from mind and need not influence working memory performance (Bocincova and Johnson, 2019; Brockhoff et al., 2022; Liao et al., 2024; Logie et al., 2011; Udale et al., 2017; Woodman et al., 2012; Xu, 2010, but see Ecker et al., 2013; Gao et al., 2016; Yin et al., 2012; Zhao et al., 2022). For instance, in a change detection paradigm, Logie et al. (2011) observed that randomizing the presentation of a task-irrelevant color feature did not influence performance at study-test intervals beyond 1 s (see also Udale et al., 2017 for a similar effect). Further, Bocincova and Johnson (2019) used pattern classification of electroencephalography (EEG) recordings and found psychophysiological evidence that processing of task-irrelevant features dissipated quickly (see also Brockhoff et al., 2022; Kerzel and Schneider, 2025). Hence, evidence from the visual short-term memory literature concordantly documents that task-irrelevant color contexts often do not influence working memory performance, especially when delays before testing exceed a second or more, as they did in the current paradigm.

Although the majority of evidence (i.e., 17 assessments) did not find support for context-dependent working memory, 2 assessments were significant in support of contextual facilitation in working memory. These two significant results may be true effects, or they may reflect Type I errors (false positives). Notably their rate of occurrence in the present study was 10.5%, not far from the standard 5% alpha level set for most statistical tests. It is also possible that the effect sizes are small, and that much larger samples would be needed to observe significant effects. Nevertheless, as the sample sizes used in the present research were similar or larger to many other long-term memory studies that observed significant context-dependent memory effects with color context manipulations (e.g., Sakai et al., 2010; Murnane and Phelps, 1995; Hockley, 2008), the present evidence indicates that context reinstatement effects are either absent or much weaker within verbal working memory.

## Data Availability

The datasets presented in this study can be found in online repositories. The names of the repository/repositories and accession number(s) can be found at: Open Science Framework: https://osf.io/yxetq/.

## Footnotes

^1^ https://websites.psychology.uwa.edu.au/school/MRCDatabase/uwa_mrc.htm

^2^For Experiment 1 only, 26 participants had 13 Match probe trials and 15 Mismatch probe trials, due to a programming error, which was corrected.

^3^A significant Shapiro–Wilk test, *p* = 0.018, indicated non-parametric tests should be used.

^4^A Shapiro–Wilk test, *p* < 0.001, indicated non-parametric tests were necessary.

^5^A nonsignificant Shapiro–Wilk test, *p* = 0.093, indicated parametric tests were warranted.

^6^A Shapiro–Wilk test confirmed that the accuracy data deviated from a normal distribution, *p* < 0.001.

^7^A Shapiro–Wilk test of normality indicated nonparametric tests were needed, *p* = 0.007.

^8^A significant Shapiro–Wilk test, *p* < 0.001, indicated the need to run nonparametric tests on working memory accuracy data.

^9^A nonsignificant Shapiro–Wilk Test indicated parametric tests were warranted, *p* = 0.169.

^10^Note that the proactive interference analysis is analogous to the response time analysis. It just more clearly quantifies the extent of proactive interference observed.

^11^A significant Shapiro–Wilk test, *p* < 0.001, indicated the need to run nonparametric tests on working memory accuracy data.

^12^A significant Shapiro–Wilk test, *p* < 0.001, indicated the need for non-parametric tests.
